# Tuning Transmission Properties of Two-Dimensional Photonic Crystal Waveguides Using Functional Dielectric Cavities

**DOI:** 10.3390/mi16050597

**Published:** 2025-05-20

**Authors:** Siqi Zhang, Feng Yang, Wenying Zhang, Wei Zhao, Luhe Yang, Hong Li

**Affiliations:** 1 Institute for Interdisciplinary Quantum Information Technology, Jilin Engineering Normal University, Changchun 130052, China; siqizhang88@163.com (S.Z.); zwyzwy8866@163.com (W.Z.); zhaow168@nenu.edu.cn (W.Z.);; 2 Jilin Engineering Laboratory for Quantum Information Technology, Changchun 130052, China; 3 School of Data Science and Artificial Intelligence, Jilin Engineering Normal University, Changchun 130052, China

**Keywords:** two-dimensional photonic crystals, photonic bandgap, defect mode, electric field distribution, transmissivity

## Abstract

In this study, the photonic band structure, transmissivity, and electric field distribution of a two-dimensional photonic crystal coupled waveguide structure are calculated using the supercell technique and finite element method. The waveguide consists of circular $KNbO_{3}$ and functional dielectric cylinders embedded in air. The dielectric constant of a functional medium cylinder is spatially dependent, which is realized through the electro-optic and Kerr effects. The dielectric constant function is defined as $\varepsilon_{c}\left(r\right)=k\cdot r+b$ ($0⩽r⩽r_{c}$), where the coefficient *k* and parameter *b* can be adjusted by an external electric field. By tuning *k* and *b*, the transmission characteristics of the waveguide, including the propagation direction and light field distribution, exhibit significant adjustability. Specifically, parameter *b* enhances or suppresses the transmissivity at output ports 1 and 2. By utilizing the regulatory capability of functional media on waveguide transmission characteristics, optical filters with specific filtering functions can be designed. These findings provide novel design strategies for advanced optical devices.

## 1. Introduction

The increasing sophistication of nanofabrication has led to the availability of highly advanced optical structures on photonic chips. The incorporation of metasurfaces and metamaterials with subwavelength structures into the fundamental components of optical waveguides is progressively transforming the field of photonic integrated circuits, resulting in a variety of meta-waveguides that exhibit exceptional capabilities in manipulating guided electromagnetic waves [1]. Recent advancements in nanofabrication techniques have markedly enhanced the manufacturing of two-dimensional photonic crystals (2D PCs), thereby facilitating their integration into optical systems.

Two-dimensional PCs [2,3,4,5] are artificial microstructured materials characterized by the periodic arrangement of dielectric materials in a two-dimensional plane. A defining feature of these materials lies in their photonic bandgap (PBG), which can prevent the propagation of light within specific frequency ranges, making them excel in wavelength-division multiplexing. Photonic crystal waveguides serve as the core components for optical wave transmission. By designing defect structures (such as point defects, line defects, or ring resonators), efficient filtering and separation functions for specific wavelengths can be achieved. Resonant cavities [6,7] have emerged as critical elements for dynamically controlling waveguide transmission characteristics by modifying structural parameters or operating conditions. The performance of traditional 2D PC waveguides relies heavily on structural parameters, such as lattice constants and filling ratios, which offer limited tunability. However, the increasing demand for broader bandwidth, lower loss, and greater flexibility in optical communication and computing applications necessitates the development of advanced waveguides. The incorporation of nonlinear materials and the optimization of cavity–waveguide coupling further enhance the performance of optical switches and sensors [8].

Potassium niobate crystal ($KNbO_{3}$) [9,10] is a strongly ferroelectric perovskite-type crystal developed in the late 1960s. Like lithium niobate [11,12,13,14], potassium niobate is also an extremely important alkali metal niobate material. It possesses particularly outstanding nonlinear optical coefficients [15,16] and exhibits excellent electro-optic, nonlinear, and piezoelectric properties. Therefore, it is one of the most commonly used materials in integrated optics and ultrasonic applications. The material’s substantial electro-optic coefficient facilitates dynamic refractive index modulation [17] through external fields, making it particularly suitable for optical modulator and switch applications. Furthermore, its pronounced second-order nonlinear optical susceptibility enables efficient frequency conversion processes, including second-harmonic generation [18].

Potassium niobate exhibits excellent ferroelectric properties, optoelectronic properties [19], large electromechanical coupling coefficients, low dielectric constants, and a high photorefractive factor Q, making it an ideal piezoelectric material to replace lead zirconate titanate (PZT). It also holds significant application value in optical storage, laser frequency doubling conversion, and acoustic wave sensors. With high chemical stability and environmental friendliness, research on potassium niobate is of great importance from both application and environmental protection perspectives. Traditional nanofabrication techniques (such as sputtering and evaporation lift-off) are prone to introducing lattice defects due to high temperatures or mechanical stress, leading to significant degradation in electro-optic coefficients. To address this, various high-precision fabrication methods have been developed in recent years to preserve the crystalline integrity of $KNbO_{3}$ [20]. For instance, the van der Waals heterointegration method leverages weak van der Waals forces to transfer pre-grown $KNbO_{3}$ nanofilms onto photonic crystal substrates, avoiding direct etching-induced damage. In the field of nanomanufacturing technology, high-quality $KNbO_{3}$ nanopillars can be grown using molecular beam epitaxy. By adjusting deposition parameters such as temperature and rate, the pillar radius can be precisely controlled. Despite the challenges in $KNbO_{3}$ nanofabrication, molecular beam epitaxy and van der Waals integration techniques have been successfully applied in the preparation of high-performance electro-optic devices [21].

This study innovatively introduces a functional dielectric cylinder with permittivity varying spatially and proposes the integration of functional dielectric cavities with electro-optic modulation to achieve tunable transmission characteristics in 2D PC waveguides. This spatially dependent permittivity property fundamentally differs from that of regular dielectrics with constant permittivity. The performance of two-dimensional regular photonic crystal (2D RPC) waveguides heavily relies on structural parameters such as lattice constants and filling ratios, offering limited tunability. The introduction of functional dielectrics enables the dynamic modulation of waveguide transmission properties by adjusting the coefficient *k* and the parameter *b* through external electric fields. Compared to traditional design schemes, this study incorporates a spatially dependent electro-optic modulation mechanism (linearly varying permittivity distribution), allowing dynamic control of optical field transmission and localized states without structural reconfiguration. This design features reusability and tuning flexibility, significantly reducing fabrication costs while meeting diverse dynamic control requirements. Such a modulation approach provides novel concepts and methodologies for the design and application of photonic crystal waveguides.

Using the supercell method combined with finite element analysis, we systematically investigate the photonic band structure (PBS), electromagnetic field distribution, and propagation characteristics of a 2D PC coupled waveguide structure. The proposed design involves the vertical removal of three rows of dielectric pillars from the 2D PC waveguide structure, followed by the insertion of four dielectric pillars to form a resonant cavity and establish a coupling configuration. The four inserted medium pillar supports are composed of functional dielectric materials, while the other medium pillars are made of a regular dielectric material, $KNbO_{3}$. Unlike regular dielectric materials with constant permittivity, functional materials exhibit position-dependent permittivity characteristics [22,23,24], where their dielectric constant is modulated through electro-optic and Kerr effects. This study specifically examines the defect mode behavior in a 2D PC coupled waveguide structure composed of $KNbO_{3}$ and functional dielectric cylinders, which have circular cross-sections. These components are arranged in a square lattice within an air matrix. This manuscript is organized as follows. Section 2 details the fundamental equations governing the PBS calculations. Section 3 presents the realization method for two-dimensional functional photonic crystals. Section 4 showcases the numerical results, demonstrating the transmissivity properties of the 2D PC structure controlled by the functionally resonant cavity. Finally, Section 5 provides the concluding remarks and discusses potential applications.

## 2. Theoretical Model

The eigenvalue equation for the TE wave, derived using the plane-wave expansion method, can be expressed as

(1) $$\begin{array}{c} \sum_{\vec{G^{\prime }}}|\vec{k}+{\vec{G}}^{\prime }\left|\right|\vec{k}+\vec{G}|\varepsilon^{-1}\left(\vec{G}-\vec{G^{\prime }}\right)E_{\vec{k}}\left(\vec{G^{\prime }}\right)=\frac{\omega^{2}}{c^{2}}E_{\vec{k}}\left(\vec{G}\right), \end{array}$$

where $\vec{k}$, $\vec{G}$, and ω represent the Bloch wave vector, reciprocal lattice vector, and angular frequency, respectively. Here, ${\vec{G}}^{\prime }$ denotes the vector of the reciprocal of the relative permittivity in the reciprocal space, *c* represents the speed of light, and $E_{\vec{k}}$ corresponds to the electric field associated with the wave vector $\vec{k}$. A detailed and comprehensible derivation process is provided in ref. [22].

The Fourier transform of the dielectric constant for two-dimensional functional photonic crystals (2D FPCs) is given by

(2) $$\begin{array}{c} \varepsilon^{-1}\left({\vec{G}}_{\left|\right|}\right)=\left\{\begin{array}{c} \frac{1}{\varepsilon_{b}}\left(1-f\right)+\frac{2f}{r_{a}^{2}}\int_{0}^{r_{a}}r\frac{1}{\varepsilon_{a}\left(r\right)}dr\;\left({\vec{G}}_{\left|\right|}=0\right)\\ \frac{2f}{r_{a}^{2}}\int_{0}^{r_{a}}r\frac{1}{\varepsilon_{a}\left(r\right)}J_{0}\left(G_{\left|\right|}\cdot r\right)dr-\frac{2f}{\varepsilon_{b}}\frac{J_{1}\left(G_{\left|\right|}\cdot r_{a}\right)}{G_{\left|\right|}\cdot r_{a}}\;\left({\vec{G}}_{\left|\right|}\neq 0\right) \end{array}\right., \end{array}$$

where $\varepsilon_{b}$ is the permittivity of air, $\varepsilon_{a}\left(r\right)$ is the position-dependent permittivity, $r_{a}$ is the radius of the medium column, $f=\frac{\pi r_{a}^{2}}{V_{0}^{\left(2\right)}}$ is the filling ratio, $V_{0}^{\left(2\right)}$ represents the unit cell area in the two-dimensional lattice space, and $J_{0}\left(G_{\left|\right|}\cdot r\right)$ and $J_{1}\left(G_{\left|\right|}\cdot r_{a}\right)$ are the Bessel functions.

When $\varepsilon_{a}\left(r\right)=\varepsilon_{a}$, where $\varepsilon_{a}$ is a constant, Equation (2) simplifies to

(3) $$\begin{array}{c} \varepsilon^{-1}\left({\vec{G}}_{\left|\right|}\right)=\left\{\begin{array}{c} \frac{1}{\varepsilon_{b}}+\left(\frac{1}{\varepsilon_{a}}-\frac{1}{\varepsilon_{b}}\right)f\;\left({\vec{G}}_{\left|\right|}=0\right)\\ 2f\left(\frac{1}{\varepsilon_{a}}-\frac{1}{\varepsilon_{b}}\right)\frac{J_{1}\left(G_{\left|\right|}\cdot r_{a}\right)}{G_{\left|\right|}\cdot r_{a}}\;\left({\vec{G}}_{\left|\right|}\neq 0\right) \end{array}\right.. \end{array}$$

Equation (3) represents the Fourier transform of the dielectric constant for 2D RPCs. Thus, 2D RPCs can be considered a specific case of 2D FPCs.

The defining characteristic of 2D FPCs is that the dielectric constant of the dielectric column varies as a function of the spatial coordinates. This is achieved by utilizing photorefractive nonlinear optical effects or electro-optic effects [25,26], which modulate the dielectric constant of the medium column based on the spatial coordinates, thereby forming 2D FPCs.

In the context of the first-order electro-optic effect, the dielectric constant function for the dielectric column of a line defect is expressed as [24]

(4) $$\begin{array}{c} \varepsilon_{r}=n_{0}^{2}+2\alpha E, \end{array}$$

where $n_{0}$ represents the refractive index in the absence of an electric field, α is the electro-optic coefficient, and *E* denotes the applied electric field.

In accordance with Faraday’s law of electromagnetic induction, the applied electric field exhibits a linear distribution along the radius *r* of the dielectric column, given by

(5) $$\begin{array}{c} E=E_{0}+dr, \end{array}$$

where $E_{0}$ is the amplitude of the electric field and *d* is a parameter.

Consequently, the dielectric constant can be reformulated as

(6) $$\begin{array}{c} \varepsilon_{r}=2\alpha dr+n_{0}^{2}+2\alpha E_{0}=kr+b. \end{array}$$

From this, we derive

(7) $$\begin{array}{c} k=2\alpha d,b=n_{0}^{2}+2\alpha E_{0}. \end{array}$$

Here, *k* (with units of $m^{-1}$) represents a function coefficient, and *b* (dimensionless) is an adjustable parameter. When k=0, the dielectric constant of the dielectric column becomes constant, resulting in 2D RPCs. When k≠0, the dielectric constant varies linearly with the radius *r*, forming 2D FPCs. By altering the intensity and distribution of the applied electric field, the function coefficient *k* and parameter *b* can be adjusted, making the dielectric column of the 2D FPCs tunable.

## 3. Realization Method for Two-Dimensional Functional Photonic Crystals

**Figure 1:**
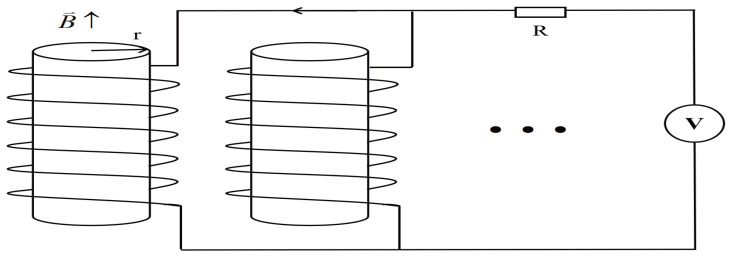
Schematic diagram of the generation of functional photonic crystals. $\vec{B}$ represents the magnetic induction intensity value in the circuit at time *t*, and its direction is along the longitudinal direction of the dielectric column. The arrow indicates the direction of the current. The bullet represent omitted medium columns.

In this section, the distribution of the external electric field is a linear function of the radius, with a symmetric distribution at the center of the scattering column as the origin column. Employing the electro-optical effect, the photonic crystals are transformed into two-dimensional functional photonic crystals. The principle is as follows: As shown in Figure 1, when both ends of all dielectric columns are connected in parallel and the external voltage is applied at the same time, the functional form of the voltage variation with time is

(8) $$\begin{array}{c} U=U_{0}t, \end{array}$$

where *U* is the voltage value in the circuit at time *t* and $U_{0}$ is the initial voltage value. Then, the current through the dielectric column is

(9) $$\begin{array}{c} i^{\prime }=i_{0}t, \end{array}$$

where $i^{\prime }$ is the current value in the circuit at time *t* and $i_{0}$ is the initial current value. According to Ampere’s loop theorem, the magnetic induction generated by the current in the dielectric column is

(10) $$\begin{array}{c} B^{\prime }=B_{0}t, \end{array}$$

where $B^{\prime }$ is the magnetic induction intensity value in the circuit at time *t* and $B_{0}$ is the initial magnetic induction intensity value.

Faraday’s law of electromagnetic induction is

(11) $$\begin{array}{c} \oint_{L}E_{i}\cdot dl^{\prime }=-\frac{d}{dt}\int_{S}B^{\prime }\cdot dS, \end{array}$$

where $E_{i}$ is the electric field strength of the induced electromagnetic field, *L* is the closed integral loop of a circle with radius *r* on the cross-section of the medium cylinder, $dl^{\prime }$ is a line element arbitrarily taken on the closed integral path *L*, and *S* is an arbitrary surface with a closed path *L* as the perimeter. From Equation (11), the induced electric current on the section of the dielectric column can be obtained.

(12) $$\begin{array}{c} E_{i}\cdot \oint_{L}dl^{\prime }=-\frac{d}{dt}\frac{d}{dt}\left[B^{\prime }\left(t\right),2\pi r^{2}\right], \end{array}$$

(13) $$\begin{array}{c} E_{i}\cdot 2\pi r=-\pi r^{2}\frac{dB^{\prime }}{dt}, \end{array}$$

(14) $$\begin{array}{c} E_{i}=-\frac{1}{2}\frac{dB^{\prime }}{dt}r=E_{0}r, \end{array}$$

where $E_{0}$ is the induced electric field amplitude, $0\leq r\leq r_{a}$. It can be seen from Equation (14) that the induced electric field strength at the section *r* of the dielectric column is proportional to *r*, known from the electro-optic effect.

(15) $$\begin{array}{c} n^{\prime }=n_{0}+m^{\prime }E=n_{0}+m^{\prime }E_{0}r=k^{\prime }r+b^{\prime }, \end{array}$$

where $n^{\prime }$ is the refractive index of the dielectric column under the external induced electric field, $k^{\prime }=m^{\prime }E_{0}$, $b^{\prime }=n_{0}$, $n_{0}$ is the refractive index when the electric field strength $E_{i}$ tends to zero, and the first-order coefficient $m^{\prime }=\frac{\alpha }{n_{0}}$, where α is a constant.

When the voltage applied to the dielectric column is changed, the electric field can be changed, and then the dielectric constant function of the dielectric column can be changed. Therefore, using this method, two-dimensional regular photonic crystals (the dielectric constant is constant) can be converted into two-dimensional functional photonic crystals (the dielectric constant is a function of the space coordinates).

## 4. Numerical Results

**Figure 2:**
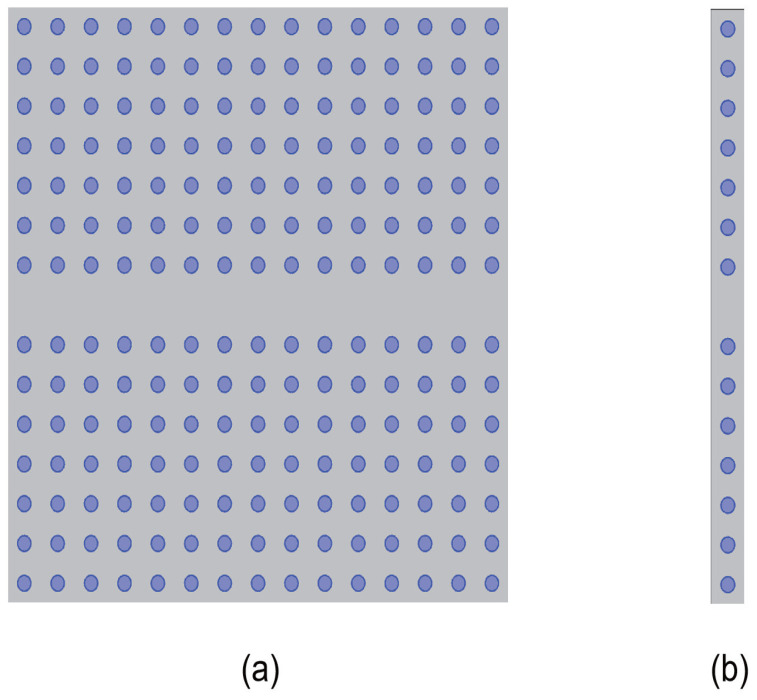
Schematic diagram of a two-dimensional photonic crystal structure. Circular dielectric columns composed of $KNbO_{3}$ ($\varepsilon_{a}=6.2$) with a radius of $r_{a}=0.2a$ are arranged in a square lattice embedded in an air medium. (**a**) Waveguide configuration; (**b**) 1 × 15 supercell configuration.

In the following analysis, we calculate the PBS, transmissivity, and electric field distribution of a 2D PC waveguide under TE polarization using COMSOL Multiphysics 4.3b. The PCs consist of circular $KNbO_{3}$ cylinders arranged in a 2D square lattice, embedded within an air background, as illustrated in Figure 2a. The central region, representing a line defect, is created by eliminating a row of dielectric columns. The spacing between these columns is set at d=2a, where $a=10^{-7}$ m represents the lattice constant. The blue columns denote conventional dielectric media with a constant dielectric constant of $\varepsilon_{a}=6.2$, while the dielectric constant of air is $\varepsilon_{b}=1$. Figure 2b depicts the supercell structure derived from Figure 2a, with an initial radius of $r_{a}=0.2a$.

**Figure 3:**
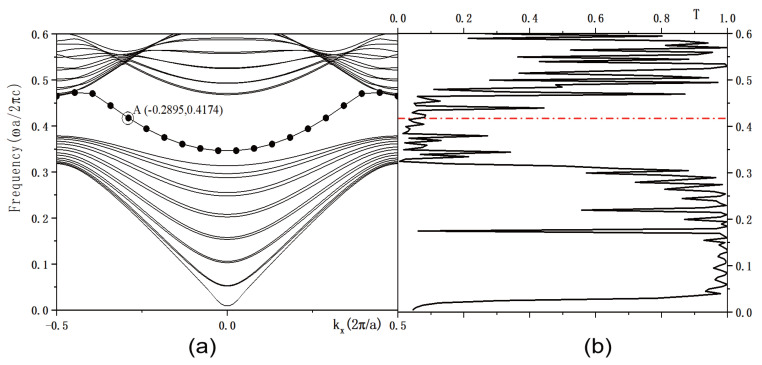
(**a**) Bandgap structure of a two-dimensional photonic crystal waveguide; (**b**) Transmissivity spectrum. The red dotted line indicates the normalized frequency of 0.4174 ωa/2πc.

**Figure 4:**
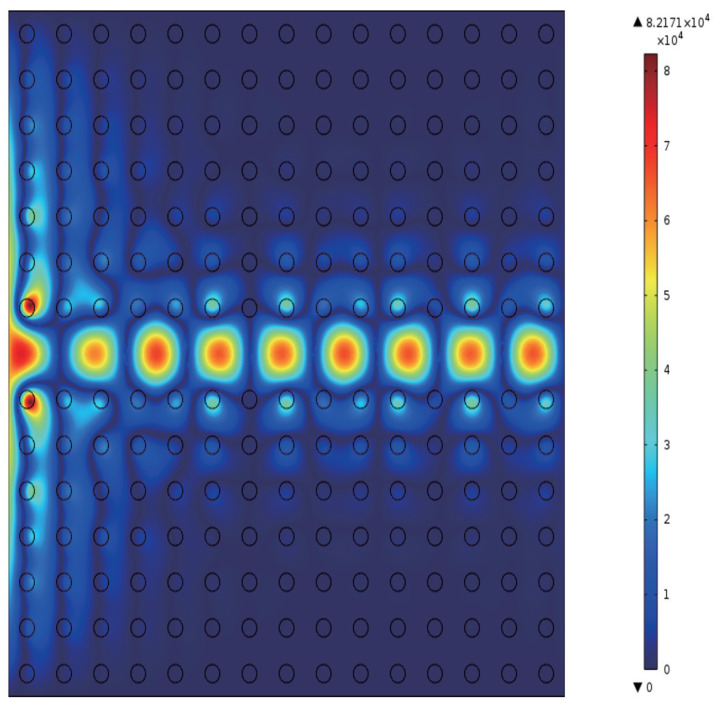
Eigenfield distribution corresponding to point A (as indicated in Figure 2a).

The calculated PBS is shown in Figure 3a. The horizontal axis denotes the wave vectors Kx in units of 2π/a, while the vertical axis indicates the normalized frequency in units of ωa/2πc. Here, ω is the angular frequency of the incident microwave, and *c* is the speed of light in vacuum. For TE polarization, a PBG is observed within the normalized frequency range of 0–0.6 (ωa/2πc). The frequency range of the bandgap is 0.3784–0.4681 (ωa/2πc), with a bandwidth of 0.09. Within this bandgap, a defect mode is identified, spanning from 0.3465 to 0.4727 (ωa/2πc), with a width of 0.126. At this defect mode, point *A* is selected, with the coordinates −0.2895, 0.4174. Figure 3b displays the transmissivity of the 2D PC waveguide. The red dotted line indicates the normalized frequency of **0.4174**ωa/2πc. The intersection of this line with the transmissivity curve corresponds to the transmissivity at point *A*, which is T=0.08. Figure 4 illustrates the electric field intensity profiles at point *A*. The right side of Figure 4 features a color gradient strip, where the color intensity indicates the field strength, increasing from bottom to top. The maximum electric field intensity reaches **$8.217\times 10^{4}$** V/m. The eigenfield is most intense within the defect region and diminishes as the distance from the defect increases.

**Figure 5:**
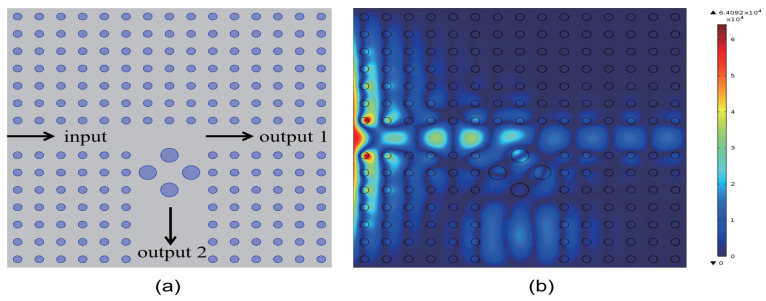
(**a**) Schematic diagram of the coupling structure between the waveguide and the conventional dielectric resonator (the dielectric constant $\varepsilon_{a}=6.2$); (**b**) Electric field distribution of the coupling structure.

**Figure 6:**
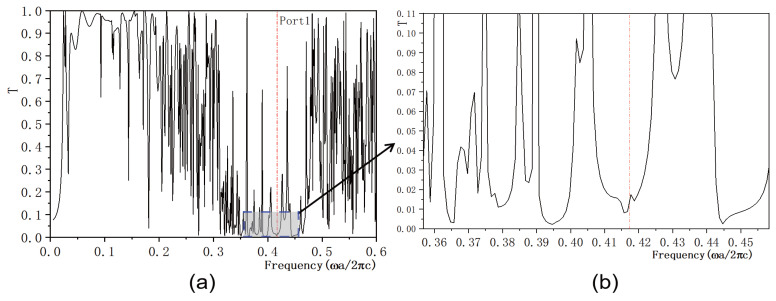
(**a**) Transmissivity curve at output 1 of the coupling structure between the waveguide and the conventional dielectric resonator; (**b**) Magnified view of the transmissivity curve. The red dotted line indicates the normalized frequency of 0.4174 ωa/2πc.

**Figure 7:**
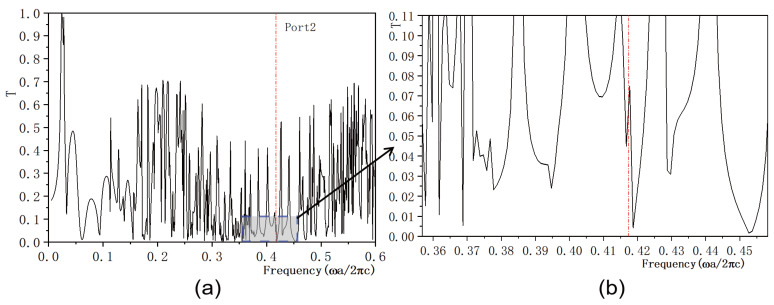
(**a**) Transmissivity curve at output 2 of the coupling structure between the waveguide and the conventional dielectric resonator; (**b**) Magnified view of the transmissivity curve. The red dotted line indicates the normalized frequency of 0.4174 ωa/2πc.

In this study, we investigate the regulation of transmissivity in a 2D PC waveguide using a functionally doped dielectric resonator cavity. In the vertical direction of the 2D PC waveguide structure, three rows of dielectric columns are removed, and four dielectric columns with a radius of $r_{c}=0.4a$ are inserted to form a microcavity. A surface light source is positioned on the left side of the waveguide, with ports 1 and 2 representing the output light in the horizontal and vertical directions, respectively. The structural layout is depicted in Figure 5a. The dielectric material of the four newly inserted columns is chosen to be the conventional dielectric ($KNbO_{3}$). The distribution of the light field at the defect mode frequency ω=0.4174ωa/2πc is illustrated in Figure 5b, where the maximum intensity reaches $6.4092\times 10^{4}$ V/m. Comparing this with Figure 4, it is evident that the introduction of a resonant cavity in the 2D PC waveguide structure results in a weakened electric field intensity, and the optical field no longer localizes at the defect position. The transmissivity curves for ports 1 is presented in Figure 6. Figure 6a displays the transmissivity curve for port 1, with the red dotted line indicating the normalized frequency of 0.4174. The transmissivity at port 1 is $T_{1}=0.017$. For clarity, an enlarged view of the transmissivity curves within the forbidden band range is provided in Figure 6b. Similarly, the transmissivity curves for ports 2 is presented in Figure 7. Figure 7a shows the transmissivity curve for port 2, with the red dotted line again marking the normalized frequency of 0.4174. The transmissivity at port 2 is $T_{2}=0.07$ ($T_{1}<T_{2}$). An enlarged view of the transmittance curves within the forbidden band range is shown in Figure 7b for better visualization. Our findings reveal that the light field intensities at both output ports 1 and 2 are significantly weak.

**Figure 8:**
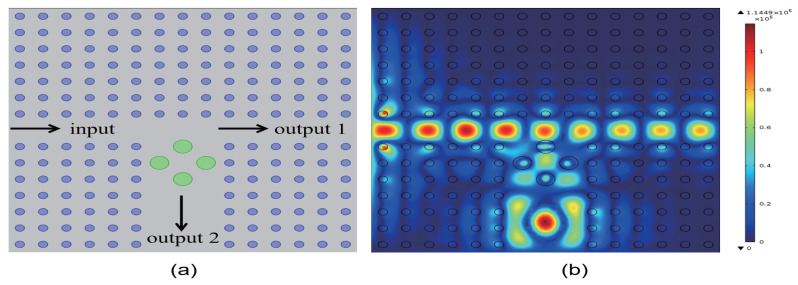
(**a**) Schematic illustration of the coupling configuration between the waveguide and the functional dielectric resonator, where the dielectric constant is defined as $\varepsilon_{c}\left(r\right)=k\cdot r+9$ ($k=4.5\times 10^{6}$ $m^{-1}$). Conventional medium columns are depicted in blue, while the functional medium column is highlighted in green; (**b**) Electric field distribution of the coupled system.

**Figure 9:**
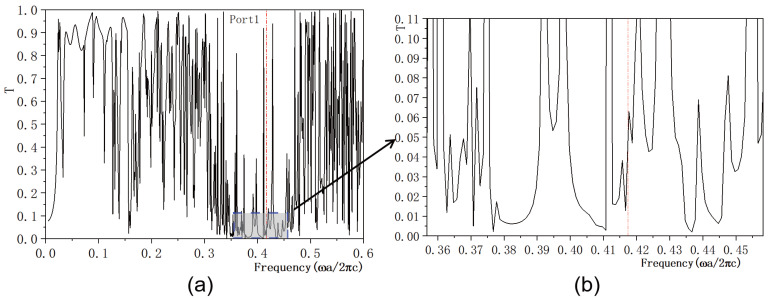
(**a**) Transmissivity curve at output 1 of the coupling structure between the waveguide and the functional dielectric resonator; (**b**) Magnified view of the transmissivity curve. The red dotted line indicates the normalized frequency of 0.4174 ωa/2πc.

Once the conventional medium column is inserted, its transmissivity and electric field distribution become fixed. To increase the transmissivity at output ports 1 and 2, a new conventional medium column would need to be reinserted, necessitating the re-preparation of the PCs. To reduce preparation costs, we propose making the dielectric constants of the four inserted medium columns a function of the spatial position, thereby creating a functional medium. By applying an electric field, light field, or magnetic field, the refractive index can be altered, enhancing the tunability of the PCs and allowing for changes in transmissivity without the need for re-preparation. A structural diagram of this design is shown in Figure 8a. The output ports are oriented in the vertical and horizontal directions. The green medium columns signify the functional medium, whose permittivity is not constant but varies with the spatial position in accordance with the form $\varepsilon_{c}\left(r\right)=k\cdot r+b$ ($0⩽r⩽r_{c}$), where *k* is the coefficient of the function and *b* is the parameter. In Figure 8b, we set the coefficient $k=4.5\times 10^{6}$ and the parameter b=9, resulting in a dielectric constant of the functional medium, given by $\varepsilon_{c}\left(r\right)=4.5\times 10^{6}\cdot r+9$. Maintaining the dielectric constant parameters of the surrounding dielectric columns as depicted in Figure 5a, the light field distribution and propagation direction of the 2D PC waveguide are illustrated in Figure 8b. The eigenfield is strongest within the defect region, with a maximum intensity reaching $1.1449\times 10^{5}$ V/m. The dielectric constant of the functional medium creates a more complex energy band structure within the defect region. This complexity enhances the localization of defect states, leading to a significant increase in the intensity of the optical field. The output light propagates in both the horizontal and vertical directions. The corresponding transmissivity curves are shown in Figure 8 and Figure 9. The transmissivity at port 1 is $T_{1}=0.062$, and at port 2, it is $T_{2}=0.175$. Compared to Figure 5b, when the dielectric constants of the four inserted dielectric columns are functions, the electric field intensity increases, and the transmissivity at output ports 1 and 2 is enhanced. The functional medium shifts the defect state frequencies closer to the edge of the PBG. This shift enhances the localization of the optical field and significantly reduces radiative loss.

**Figure 10:**
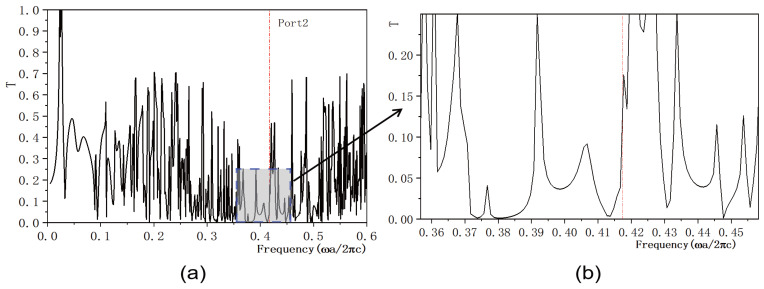
(**a**) Transmissivity curve at output 2 of the coupling structure between the waveguide and the functional dielectric resonator; (**b**) Magnified view of the transmissivity curve. The red dotted line indicates the normalized frequency of 0.4174 ωa/2πc.

**Figure 11:**
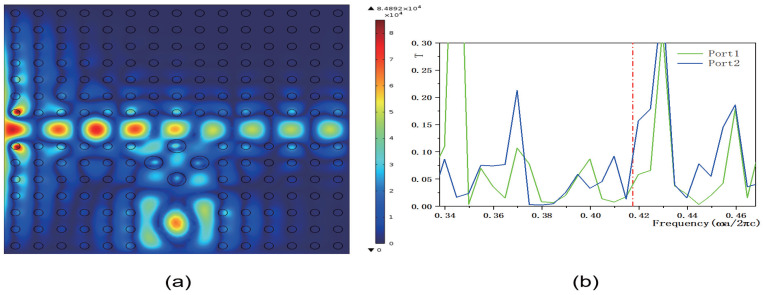
The coupling structure between the waveguide and the functional dielectric resonator (the dielectric constant $\varepsilon_{c}\left(r\right)=k\cdot r+9$, where $k=1.5\times 10^{6}$ $m^{-1}$). (**a**) Electric field distribution; (**b**) Transmissivity curves at output 1 and output 2. The red dotted line indicates the normalized frequency of 0.4174 ωa/2πc.

**Figure 12:**
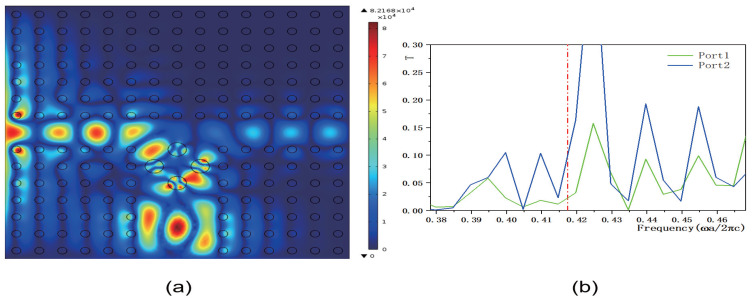
The coupling structure between the waveguide and the functional dielectric resonator (the dielectric constant $\varepsilon_{c}\left(r\right)=k\cdot r+9$, where $k=8.5\times 10^{6}$ $m^{-1}$). (**a**) Electric field distribution; (**b**) Transmissivity curves at output 1 and output 2. The red dotted line indicates the normalized frequency of 0.4174 ωa/2πc.

**Figure 13:**
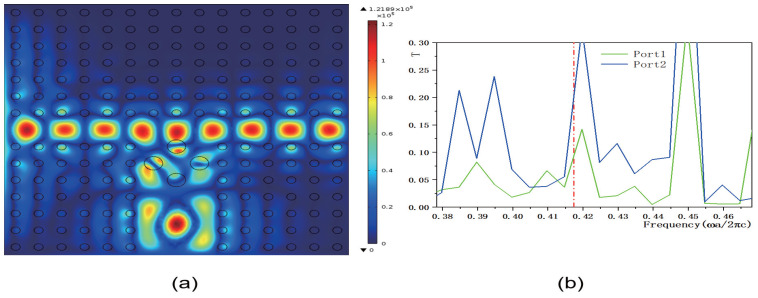
The coupling structure between the waveguide and the functional dielectric resonator (the dielectric constant $\varepsilon_{c}\left(r\right)=k\cdot r+3$, where $k=4.5\times 10^{6}$ $m^{-1}$). (**a**) Electric field distribution; (**b**) Transmissivity curves at output 1 and output 2. The red dotted line indicates the normalized frequency of 0.4174 ωa/2πc.

**Figure 14:**
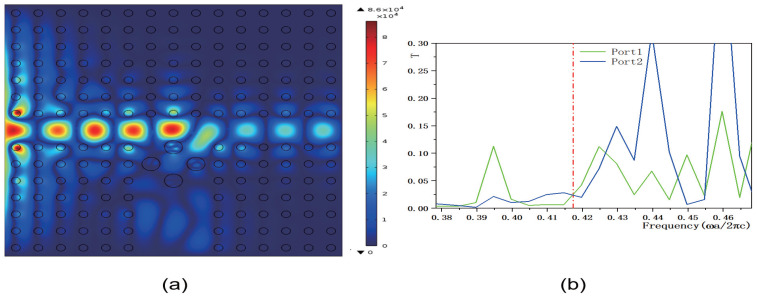
The coupling structure between the waveguide and the functional dielectric resonator (the dielectric constant $\varepsilon_{c}\left(r\right)=k\cdot r+20$, where $k=4.5\times 10^{6}$ $m^{-1}$). (**a**) Electric field distribution; (**b**) Transmissivity curves at output 1 and output 2. The red dotted line indicates the normalized frequency of 0.4174 ωa/2πc.

Although the transmissivity of output ports 1 and 2 improves, the overall transmissivity values remain relatively low. To address this, we designed a method to control the transmissivity of 2D PCs using a functionally doped dielectric resonator cavity. Figure 11, Figure 12, Figure 13 and Figure 14 illustrate how the transmissivity of the coupled structure of 2D PCs can be controlled by adjusting the values of *k* and *b*. First, we varied the value of the coefficient *k*, setting it to $k=1.5\times 10^{6}$ $m^{-1}$ and $k=8.5\times 10^{6}$ $m^{-1}$, respectively, while keeping the other parameters consistent with those in Figure 8. The corresponding electric field distributions are depicted in Figure 11a and Figure 12a, and the transmissivity curves are presented in Figure 11b and Figure 12b. The green and blue lines indicate the transmissivity of port 1 and port 2 within the normalized frequency range of 0−0.6ωa/2πc. Compared to Figure 8b, reducing the value of *k* results in a decrease in the electric field intensity. The output light propagates in both the horizontal and vertical directions. Compared to Figure 9b, the transmissivity of port 1 decreases to ($T_{1}=0.038$), and compared to Figure 10b, the transmissivity of port 2 decreases to ($T_{2}=0.09$). Conversely, increasing the value of *k* also leads to a reduction in the electric field intensity. In this case, the output light primarily travels in the vertical direction, with minimal propagation in the horizontal direction. Compared to Figure 9b, the transmissivity of port 1 decreases to $T_{1}=0.02$, with almost no light passing through, and compared to Figure 10b, the transmissivity of port 2 decreases to $T_{2}=0.09$. These results indicate that adjusting the value of *k* has an inhibitory effect on the transmissivity values of both ports. The coefficient *k* directly influences the rate of change of the dielectric constant gradient. Specifically, a larger value of *k* results in a steeper dielectric constant gradient, which, in turn, modifies the propagation path of light waves and affects the efficiency of mode coupling.

Next, we adjusted the value of the parameter *b*, setting it to b=3 and b=20, while keeping the other parameters consistent with those in Figure 8. The corresponding electric field distributions are illustrated in Figure 13a and Figure 14a, and the transmissivity curves are presented in Figure 13b and Figure 14b. Compared to Figure 8b, reducing the value of *b* increases the electric field intensity. The output light propagates in both the horizontal and vertical directions. Compared to Figure 9b, the transmissivity of port 1 increases to $T_{1}=0.1$, and compared to Figure 10b, the transmissivity of port 2 increases to $T_{2}=0.22$. Conversely, increasing the value of *b* decreases the electric field intensity. In this scenario, the output light mainly propagates in the horizontal direction. Compared to Figure 9b, the transmissivity of port 1 decreases to $T_{1}=0.025$, and compared to Figure 10b, the transmissivity of port 2 decreases to $T_{2}=0.025$. These findings suggest that adjusting the value of *b* can either enhance or weaken the transmissivity values of ports 1 and 2. The parameter *b* serves to adjust the reference value of the dielectric constant. A smaller *b* can reduce the overall dielectric constant of the dielectric column, which optimizes the PBG structure and enhances the transmission of light waves through specific ports.

**Figure 15:**
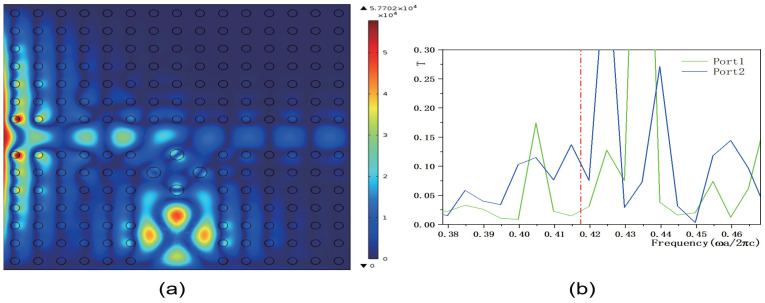
The coupling structure between the waveguide and the functional dielectric resonator (the dielectric constant $\varepsilon_{c}\left(r\right)=k\cdot r+9$, where $k=4.5\times 10^{6}$ $m^{-1}$, r(c)=0.3a). (**a**) Electric field distribution; (**b**) Transmissivity curves at output 1 and output 2. The red dotted line indicates the normalized frequency of 0.4174 ωa/2πc.

Figure 15 investigates the influence of the radius of the functional dielectric rods on the transmission characteristics of the two-dimensional photonic crystal waveguide. The radius of the functional dielectric rods is reduced from $r_{c}=0.4a$ (Figure 7a) to $r_{c}=0.3a$, while the other parameters remain the same as those in Figure 8. The electric field distribution is shown in Figure 15a, and the transmissivity curves for port 1 and port 2 are presented in Figure 15b. When the radius of the functional dielectric rods decreases, the electric field intensity weakens. The smaller radius may adjust the mode matching between the waveguide and the functional dielectric, allowing more optical energy to be guided to the output port, thereby preventing the optical energy from being effectively localized in the defect region. The transmissivity of port 1 is $T_{2}=0.025$, and that of port 2 is $T_{2}=0.11$. Although these values are lower than the transmissivity observed in Figure 9 and Figure 10, they are still stronger than the transmissivity of waveguide structures coupled with conventional dielectric materials under equivalent conditions.

When *k*, *b*, and the radius of the functional dielectric are adjusted, the light field distribution and propagation direction in the 2D PC coupled structure waveguide can be altered. This implies that by modifying *k* or *b*, we can control the light field distribution and propagation direction within the waveguide.

## 5. Conclusions

Using the supercell method and finite element analysis, we calculated the PBS, light field distribution, and propagation direction of a 2D PC coupled structure waveguide. The transmissivity of the 2D PC waveguide was regulated through the functional medium resonant cavity. Our numerical calculations yielded the following results:

1. When the dielectric constants of the four inserted dielectric columns are functions, the electric field intensity increases, and the transmissivity at output ports 1 and 2 is enhanced.
2. Adjusting the value of *k* has an inhibitory effect on the transmissivity values of ports 1 and 2.
3. Adjusting the value of *b* can either enhance or weaken the transmissivity values of ports 1 and 2.
4. The propagation direction of a 2D PC coupled structure waveguide can be controlled by changing *k* and *b*.
5. When the radius of the functional dielectric cylinder decreases, the electric field intensity weakens, and the transmissivity of ports 1 and 2 decreases.

This study achieved tunability in the transmissivity and propagation direction through functional media, rather than pursuing absolute high transmissivity. Reconfigurable photonic circuits enable functional adjustments without the need for remanufacturing, significantly reducing costs. This dynamic control capability holds important value in optical switches and multiplexers.

## Data Availability

Data are contained within the article.
